# Assessment of Passive Smoking in Children With Asthma Using Urinary Cotinine Levels and Its Association With Asthma Severity

**DOI:** 10.7759/cureus.88583

**Published:** 2025-07-23

**Authors:** Dipti Agarwal, Richa Choudhary, Shamrendra Narayan, Pradeep K Yadav, Krishna K Singh

**Affiliations:** 1 Department of Pediatrics, Dr. Ram Manohar Lohia Institute of Medical Sciences, Lucknow, IND; 2 Department of Forensic Medicine and Toxicology, Dr. Ram Manohar Lohia Institute of Medical Sciences, Lucknow, IND; 3 Department of Radiodiagnosis and Imaging, Dr. Ram Manohar Lohia Institute of Medical Sciences, Lucknow, IND

**Keywords:** asthma severity, medicolegal aspects, passive smoking, pediatric asthma, urine cotinine level

## Abstract

Introduction

Passive smoking can exacerbate asthma symptoms in children. Although cotinine levels offer an accurate measure of passive smoking, their use in clinical, forensic, and medicolegal documentation remains limited. This study aimed to evaluate passive smoking in children with asthma by measuring urinary cotinine levels and to explore the forensic and medicolegal implications of documenting such exposure. The association of cotinine levels with asthma severity was examined alongside environmental and host-related factors. Cotinine levels were also correlated with demographic characteristics and parental smoking status.

Methods

Children newly diagnosed with asthma were enrolled in the study and underwent thorough clinical evaluation. To minimize confounding, children with recent respiratory infections, known environmental allergen exposure, or other chronic respiratory conditions were excluded. Parental smoking was assessed through a structured questionnaire. Urinary cotinine levels were measured using the high-performance liquid chromatography method to assess passive smoking. Host factors (age, sex, family history, and associated allergies) and environmental triggers (passive smoking, cold air, dust, seasonal variation, and residential setting) were also evaluated in relation to asthma severity.

Results

Among 92 children with asthma, 32 (34.8%) had cotinine levels within the passive smoking range. Children aged ≥6 years and those with a family history of asthma showed a significant association with asthma severity (p = 0.001 and p = 0.032, respectively). Cotinine levels within the passive smoking range were significantly correlated with disease severity (p = 0.043). Parental reporting identified only 30.3% of children exposed to passive smoking. In contrast, cotinine biomarker analysis provided objective evidence of environmental tobacco smoke exposure, underscoring the medicolegal importance of such documentation in clinical practice.

Conclusions

A considerable proportion of children with asthma demonstrated cotinine levels indicative of passive smoking. Passive smoking was significantly associated with increased asthma severity. Compared to parental reporting, urinary cotinine levels offer a more accurate assessment of passive smoke exposure. As a reliable biomarker, urinary cotinine links clinical findings with forensic documentation, reinforcing its role in medicolegal reporting. Integrating cotinine testing into clinical practice may support preventive strategies and strengthen legal advocacy for at-risk pediatric populations.

## Introduction

Asthma is the most common chronic disease in children [1]. It presents with a wide range of respiratory symptoms that vary in severity, and acute exacerbations can sometimes be fatal [2]. The severity of asthma may be influenced by multiple factors, including exposure to dust mites, molds, air pollutants, and other allergens. Among these, tobacco smoke has been a significant focus of research due to its potential to trigger and worsen asthma in children.

According to a report by the World Health Organization, more than 700 million children worldwide are exposed to passive smoking [1]. Children are particularly vulnerable to the harmful effects of passive smoke because of their narrower airways, higher respiratory rates, and underdeveloped immune systems [3]. Those with asthma are even more susceptible to its detrimental impact [4]. Passive smoke exposure in asthmatic children is linked to more frequent exacerbations and increased bronchial hyperreactivity [4]. It may also contribute to increased disease severity [5]. Reducing children’s exposure to passive smoking has been shown to aid in both the prevention and management of asthma [6].

Passive smoking refers to the involuntary inhalation of a complex mixture of tobacco smoke [7]. Cotinine, a metabolite of nicotine, is widely recognized as the most reliable biomarker for assessing tobacco smoke exposure [8]. It is preferred over nicotine itself because it remains in the body for a longer period, making it easier to detect [9]. From a forensic medicine standpoint, urinary cotinine levels serve not only as clinical indicators of environmental tobacco smoke (ETS) exposure but also as objective evidence in medicolegal cases. These may include custody disputes, child neglect investigations, or public health litigations where tobacco smoke is implicated in adverse outcomes. In such contexts, quantifiable biomarkers like cotinine offer concrete proof, bridging the gap between clinical assessment and legal accountability [10]. Thus, measuring urinary cotinine has implications that extend beyond clinical diagnosis: it supports toxicological surveillance and reinforces the legal and ethical responsibility to mitigate preventable health risks in children.

This study was conducted with the aim of quantifying urinary cotinine levels in children with asthma, correlating cotinine levels with asthma severity, and examining the relationship between parental smoking and urinary cotinine levels. It also sought to highlight the medicolegal implications of objectively documenting passive smoke exposure. Additional correlations were made between asthma severity and other environmental and host-related factors, as well as between cotinine levels, demographic characteristics, and reported caregiver smoking.

## Materials and methods

This cross-sectional study was conducted in the Departments of Pediatrics and Forensic Medicine and Toxicology at Dr. Ram Manohar Lohia Institute of Medical Sciences in Lucknow, India, following approval from the Institutional Ethics Committee. Participant recruitment took place between March 2020 and December 2021. Children aged 3 to 12 years with a clinical diagnosis of asthma who attended the pediatrics department were enrolled after obtaining informed consent from their caregivers. In addition, assent was obtained from children aged over 7 years and under 18 years. The diagnosis of asthma was made according to the Global Initiative for Asthma (GINA) guidelines [11], which define asthma as a condition characterized by variable respiratory symptoms - such as wheezing, shortness of breath, chest tightness, and coughing - that vary in intensity and over time [10,11]. Lung function testing using spirometry was performed in children aged over 6 years. Children who were already receiving asthma treatment or had other chronic respiratory diseases were excluded to avoid potential confounding from medications (e.g., corticosteroids or bronchodilators) that could affect symptom severity, inflammatory responses, or cotinine metabolism.

The sample size was calculated using a prevalence (p) of asthma of 37.5%, an allowable error (l) of 10%, and a 5% level of significance (z = 1.96) [12]. Using the formula n = z² × p(1-p) / l², the required sample size was estimated to be 91.4, which was rounded up to 92 cases.

Enrolled children underwent a comprehensive evaluation that included both host factors (age, gender, family history of atopic disease, and associated allergies) and environmental triggers (exposure to cold food or air, dust, respiratory infections, seasonal changes, and other relevant factors). A family history was considered positive if either parent had a consistent history of atopic conditions such as asthma, rhinitis, or eczema.

Exposure to passive smoking was assessed using a simple questionnaire administered to caregivers. The questionnaire included the following items: (1) whether the caregiver had smoked within the last three days (yes/no); (2) whether smoking occurred inside the home (never/sometimes/always); and (3) whether smoking occurred in the presence of the child (never/sometimes/always). A child was considered exposed to passive smoking if a “yes” or nonzero response was given to any of the three domains, indicating the presence of a smoker in the household, smoking in enclosed spaces, or direct exposure in the child’s presence. Children whose responses were “no” or “never” in all domains were considered unexposed.

The severity of asthma in the study participants was classified into intermittent, mild persistent, moderate persistent, and severe persistent asthma. This classification was based on the frequency of symptoms, nighttime awakenings, the degree of interference with normal daily activities, and lung function test results. Intermittent asthma was defined as having symptoms on two or fewer days per week, nighttime awakenings two or fewer times per month, no limitation of normal activity, and an FEV1/FVC ratio greater than 85%. Mild persistent asthma was characterized by symptoms occurring more than two days per week but not daily, nighttime awakenings three to four times per month, minor activity limitations, and an FEV1/FVC ratio equal to or greater than 80%. Moderate persistent asthma was defined by daily symptoms, nighttime awakenings more than once per week but not nightly, some activity limitation, and an FEV1/FVC ratio between 75% and 80%. Severe persistent asthma was defined by symptoms occurring throughout the day, nighttime awakenings as often as seven times per week, extremely limited daily activity, and an FEV1/FVC ratio below 75%.

Approximately 2 mL of urine was collected from each subject and transported to the Department of Forensic Medicine and Toxicology for the estimation of cotinine levels. Cotinine analysis was performed using high-performance liquid chromatography. The procedure utilized a Chromasil C18 column (150 mm × 4.6 mm i.d.) and an isocratic mobile phase consisting of phosphate buffer and acetonitrile in an 83:17 volume ratio. The phosphate buffer (0.02 M) contained 0.1% triethylamine, and the pH was adjusted to 6.72 using orthophosphoric acid. The flow rate was maintained at 0.7 mL per minute. The method was sensitive enough to detect cotinine concentrations as low as 0.1 mg/L. Cotinine levels below 10 ng/dL were considered detectable [13]. Levels between 10 and 50 ng/dL were categorized as indicative of passive smoking, while levels exceeding 50 ng/dL were interpreted as active smoking [13].

Statistical analysis

The data were entered into a Microsoft Excel spreadsheet (Microsoft Corporation, Redmond, WA, USA) for analysis. For statistical comparison, children with mild persistent and intermittent asthma were grouped together as the mild asthma group, while those with moderate and severe persistent asthma were categorized as the severe group. The associations between asthma severity and cotinine levels, host factors, and environmental triggers were assessed using the chi-square test for nonparametric data and the Mann-Whitney U test for parametric data. Cotinine levels in the negative and detectable ranges were grouped and compared against levels indicative of passive smoking to examine associations with demographic variables (age, gender, and rural/urban residence) and parental smoking status. These comparisons were performed using the Kruskal-Wallis test. A p-value of <0.05 was considered statistically significant, while a p-value of <0.001 was considered highly significant.

## Results

During the study period, a total of 92 children with asthma were enrolled. Of these, 45 cases (48.9%) were classified as having mild asthma, while 47 cases (51.1%) were categorized as moderate to severe. The majority of participants were older than 6 years, accounting for 57 cases (62%). Male children predominated, with 70 cases (76%), compared to female participants. Cotinine levels within the passive smoking range were observed in 32 children (34.8%), with a mean cotinine concentration of 17.53 ng/dL (Table 1).

**Table 1:** Cotinine levels in study subjects with asthma (n = 92)

Cotinine level	Frequency n (%)	Mean (ng/dL)	SD	95% CI	Range (ng/dL)
Negative	27 (29.3%)	0	0	0	0
Detectable	33 (35.9%)	1.49	± 1.37	(1.01, 1.98)	0.5-5
Passive	32 (34.8%)	17.53	± 12.72	(12.94, 22.11)	10-64
Total	92 (100%)	6.63	± 10.96	(4.36, 18.90)	0-64

The correlation between host factors and asthma severity is presented in Table 2. A significantly higher number of children aged ≥6 years were in the severe asthma group (37 cases, 78.7%), whereas mild disease was more common in children aged <6 years (25 cases, 55.5%) (p = 0.001). There was no statistically significant difference between the two groups in terms of gender distribution (p = 0.711) or the presence of atopic conditions (p = 0.104). However, a significant association was observed between family history and asthma severity, with 34 children (72.3%) in the severe group having a positive family history of atopic disease (p = 0.032).

**Table 2:** Correlation of host factors with asthma severity ^*^ Significant ^**^ Highly significant

S no.	Variables		Total cases (92), n (%)	Mild cases (45), n (%)	Moderate/severe cases (47), n (%)	χ²	p-Value
1	Age	<6 years	35 (38)	25 (55.5)	10 (21.3)	11.460	0.001^**^
		≥6 years	57 (62)	20 (45.5)	37 (78.7)		
2	Gender	Female	22 (34)	10 (22.2)	12 (25.5)	0.138	0.711
		Male	70 (76)	35 (77.8)	35 (74.5)		
3	Family history	Yes	56 (60.8)	22 (48.8)	34 (72.3)	5.308	0.032^*^
		No	33 (39.2)	23 (51.2)	13 (27.7)		
4	Associated allergies	Yes	30 (32.6)	11 (24.4)	19 (40.4)	2.672	0.104
		No	62 (67.4)	34 (75.6)	28 (59.6)		

The relationship between environmental triggers and asthma severity is summarized in Table 3. Passive smoking was significantly associated with more severe asthma, observed in 21 children (44.7%) in the severe group compared to 11 children (24.4%) in the mild group (p = 0.043). Other environmental factors - including seasonal variation (p = 0.725), residence (urban vs. rural; p = 0.291), exposure to cold air or food (p = 0.307), dust (p = 0.479), and respiratory infections (p = 0.895) - did not show a significant association with asthma severity. 

**Table 3:** Association of cotinine levels and environmental factors with asthma severity p < 0.05 is considered significant.

S. no.	Variable		Total, n (%)	Mild severity (45), n (%)	Moderate/severe severity (47), n (%)	χ²	p-Value
1	Cotinine levels	Passive smoking	32 (34.8)	11 (24.4)	21 (44.7)	4.150	0.043
		Detectable/negative	60 (65.2)	34 (75.6)	26 (55.3)		
2	Residence	Rural	50 (54.3)	27 (60.0)	23 (49.0)	1.134	0.291
		Urban	42 (45.7)	18 (40.0)	24 (51.0)		
3	Seasonal variation	Yes	24 (26.0)	11 (24.4)	16 (34.0)	0.123	0.725
		No	68 (74.0)	34 (75.6)	31 (66.0)		
4	Cold air	Yes	46 (50.0)	20 (44.4)	26 (55.3)	1.088	0.307
		No	46 (50.0)	25 (55.6)	21 (44.7)		
5	Dust	Yes	17 (18.5)	7 (15.6)	10 (21.3)	0.450	0.479
		No	75 (81.5)	38 (84.4)	37 (78.7)		
6	Respiratory infections	Yes	15 (16.3)	13 (28.9)	13 (27.6)	0.0171	0.895
		No	77 (83.7)	32 (71.1)	34 (72.4)		
7	Others	Yes	14 (15.2)	5 (11.1)	9 (19.1)	0.060	0.806
		No	78 (84.8)	40 (88.9)	38 (80.9)		

As shown in Table 4, passive smoking levels of cotinine were more frequently observed in children aged >6 years, with 24 cases (75%), compared to only 8 cases (25%) among children aged <6 years (p = 0.061). There was no statistically significant difference in cotinine levels based on gender. Similarly, no significant association was found between cotinine levels and the place of residence (urban vs. rural). Among children with cotinine levels in the passive smoking range, 18 (56.2%) resided in rural areas and 14 (48.8%) in urban areas (p = 0.799). A significant correlation was observed between cotinine levels and reported smoking among parents or caregivers (p = 0.001). However, parental reporting identified only 10 cases (31.3%) of children with passive smoke exposure, highlighting the limited sensitivity of self-reported smoking.

**Table 4:** Cotinine levels in relation to demographic profile and parental smoking p < 0.001 is considered highly significant.

Variables		Negative/detectable smoking level (60), n (%)	Passive smoking level (32), n (%)	χ²	p-Value	OR (CI)
Age	<6 years	27 (45)	8 (25)	3.542	0.061	2.45 (0.95-1.33)
	≥6 years	33 (55)	24 (75)			
Gender	Female	16 (26.6)	6 (18.8)	0.719	0.399	1.57 (0.55, 4.33)
	Male	44 (73.4)	26 (81.2)			
Residence	Rural	32 (53.4)	18 (56.2)	0.072	0.799	0.89 (0.37, 2.10)
	Urban	28 (46.6)	14 (48.8)			
Parental Smoking	Yes	1 (1.6)	10 (31.3)	17.35	0.0001	30.73 (20.60,5 0.33)
	No	59 (98.4)	22 (68.7)			

Medicolegal relevance

Urinary cotinine testing objectively confirmed passive smoking exposure in 34.8% of children, whereas caregiver self-reporting identified only 30.3%, underscoring the forensic value of biomarker-based evidence in medicolegal documentation. The significant association between elevated cotinine levels and severe asthma (p = 0.043) further reinforces the clinical and legal relevance of this biomarker. Objective reporting through urinary cotinine analysis can play a critical role in child protection cases, support legal actions involving environmental harm, and inform public health policymaking. Incorporating cotinine testing into medicolegal assessments provides a robust scientific foundation for protecting vulnerable pediatric populations from the harmful effects of passive smoking (Table 5).

**Table 5:** Medicolegal relevance of urinary cotinine analysis in pediatric asthma cases

Aspect	Findings	Medicolegal importance
Detection by urinary cotinine	34.8% of cases detected	Provides objective proof of passive smoking exposure
Detection by self-reported smoking	30.3% of cases detected	Highlights underreporting and emphasizes the need for biomarker validation
Association with severity of asthma	Significant association (p = 0.043)	Supports clinical impact and justifies medicolegal documentation and reporting
Demographic influence (gender and residence)	No significant association	Indicates widespread exposure, reinforcing the need for child protection measures
Child protection implications	Elevated cotinine linked to increased asthma severity	Establishes legal grounds for intervention in cases of environmental harm

## Discussion

Passive smoking is a well-known trigger for asthma symptoms; however, its association with asthma severity in children remains underexplored. This study aimed to investigate the relationship between asthma severity and urinary cotinine levels, a reliable biomarker of tobacco smoke exposure in children. In our study, 34.8% of asthmatic children had urinary cotinine levels within the range indicative of passive smoking. A similar finding was reported by Tovar et al., where 33.96% of children admitted with respiratory disorders showed positive urine cotinine levels ranging from 10 to 100 ng/dL [13]. In our cohort, urinary cotinine ranged from 0 to 64 ng/dL. Another study reported cotinine levels ranging from undetectable to 134 ng/dL [14]. These findings support the strong association between asthma severity and urinary cotinine levels observed in our study.

We also examined the relationship between asthma severity and various host factors. Children older than 6 years were significantly more likely to have severe disease. In contrast, Hassanzad et al. found no significant association between age and asthma severity [15]. While asthma was more prevalent among males in our study, the association between gender and disease severity was not statistically significant, aligning with findings from previous research [16]. We also found that other atopic conditions did not significantly correlate with asthma severity, although another study reported a significant association between allergic rhinitis and severe asthma [17]. In our study, a positive family history of atopic disease was significantly associated with more severe asthma, consistent with findings from other research showing higher rates of moderate to severe asthma among children with a family history of atopy [18].

Tobacco smoke exposure is a well-established risk factor for childhood asthma [19], yet only a limited number of studies have assessed this exposure using biochemical markers like cotinine [20]. Cotinine remains the most accurate biomarker for evaluating passive smoking exposure [21]. In our study, higher urinary cotinine levels were significantly associated with increased asthma severity. A comparable study reported a significant association between urine cotinine levels and risk of severe asthma (odds ratio 3.56, CI = 1.29-5.53, p = 0.01) [15]. Elevated urinary cotinine levels have also been reported in children presenting with coughing, rhinorrhea, and sneezing compared to those without respiratory symptoms [21]. Another study found that children with asthma who were exposed to passive smoke exhibited greater bronchial reactivity, more frequent emergency room visits, and reduced lung function [19]. Additionally, higher urinary cotinine levels were positively associated with the number of asthma symptom days in children [15].

We also examined the association between cotinine levels and demographic variables. In our study, a significant proportion of children with passive smoking levels of cotinine were older than 6 years. However, another study did not report such an age-related association [15]. One possible explanation for this finding in our study is that older children may be more frequently exposed to smoking environments, such as workplaces or public spaces, or in rare cases may have begun smoking themselves. No gender-related differences in cotinine levels were observed in our study, consistent with previous research showing no significant difference in passive smoking exposure between males and females [15]. The place of residence (urban vs. rural) also showed no significant association with cotinine levels. A similar lack of statistical significance was observed in a study of adolescents, where 59.62% of urban residents and 40.38% of rural residents showed cotinine levels indicative of passive smoking [22].

Children are most commonly exposed to tobacco smoke from smoking by parents or caregivers at home. While self-reporting by parents is a convenient and low-cost method of assessing exposure, it is often unreliable due to underreporting or denial of smoking behavior [23]. In our study, passive smoking was also assessed through parental questionnaires, which identified fewer cases than those detected through urinary cotinine analysis. Similar findings have been reported in other studies [24]. Cases where cotinine was detected without documented caregiver smoking may be attributed to environmental exposure to residual tobacco smoke, as suggested in earlier research [14].

The detection of urinary cotinine in children with asthma has significant implications beyond clinical assessment. It provides a basis for medicolegal evaluation of environmental health risks. Quantifying cotinine enables clinicians not only to assess exposure but also to generate objective, legally admissible evidence in cases involving environmental negligence [25]. In forensic toxicology, particularly in child protection or public health litigation, the use of urinary cotinine to document exposure to ETS can substantiate claims of parental or institutional neglect. This is especially relevant in high-risk populations where repeated hospital admissions for asthma exacerbations may be attributed to modifiable environmental exposures [26]. Furthermore, objective biomarker evidence can aid in enforcing indoor smoking regulations, strengthening smoke-free housing policies, and highlighting the public health responsibility associated with preventing passive smoking.

Our study adds to the growing body of evidence by reinforcing the association between passive smoke exposure and asthma severity through a biochemical marker in an Indian pediatric population. It emphasizes the need to integrate objective exposure assessments into both clinical and forensic practice. Thus, this study underscores the dual utility of urinary cotinine as a clinical biomarker and a forensic indicator, enhancing disease management while supporting accountability for toxic environmental exposures in children.

Limitations

The study had a few limitations. Being a pilot study, the sample size was relatively small and calculated based on the known prevalence of asthma. The cross-sectional design limits causal inference. Recall bias may have affected the accuracy of parental reporting, and the single-center setting may introduce selection bias. Additionally, potential confounding factors, such as indoor pollution and ventilation status, were not assessed. The location of smoking (indoor vs. outdoor) was also not specifically defined.

## Conclusions

The study reveals a concerningly high proportion of children with asthma who have urinary cotinine levels indicative of passive smoking exposure. Our findings underscore a significant association between passive smoking and the severity of asthma in children. Among the host factors, older age and a positive family history were significantly linked with more severe forms of the disease. The study also highlights the value of urinary cotinine assessment as a more accurate and objective measure of tobacco smoke exposure compared to parental self-reporting. In addition to informing effective clinical management, these findings point to the need for further research into the underlying causes of pediatric asthma. Most importantly, the results should serve as a wake-up call to the healthcare community to strengthen public health initiatives aimed at shielding children from the harmful effects of ETS.
